# Impaired discourse content in aphasia is associated with frontal white matter damage

**DOI:** 10.1093/braincomms/fcad310

**Published:** 2023-11-10

**Authors:** Junhua Ding, Erica L Middleton, Daniel Mirman

**Affiliations:** Department of Psychology, University of Edinburgh, Edinburgh EH8 9JZ, UK; Moss Rehabilitation Research Institute, Elkins Park, PA 19027, USA; Department of Psychology, University of Edinburgh, Edinburgh EH8 9JZ, UK

**Keywords:** speech production, aphasia, communication, picture description, communication efficiency

## Abstract

Aphasia is a common consequence of stroke with severe impacts on employability, social interactions and quality of life. Producing discourse-relevant information in a real-world setting is the most important aspect of recovery because it is critical to successful communication. This study sought to identify the lesion correlates of impaired production of relevant information in spoken discourse in a large, unselected sample of participants with post-stroke aphasia. Spoken discourse (*n* = 80) and structural brain scans (*n* = 66) from participants with aphasia following left hemisphere stroke were analysed. Each participant provided 10 samples of spoken discourse elicited in three different genres, and ‘correct information unit’ analysis was used to quantify the informativeness of speech samples. The lesion correlates were identified using multivariate lesion–symptom mapping, voxel-wise disconnection and tract-wise analyses. Amount and speed of relevant information were highly correlated across different genres and with total lesion size. The analyses of lesion correlates converged on the same pattern: impaired production of relevant information was associated with damage to anterior dorsal white matter pathways, specifically the arcuate fasciculus, frontal aslant tract and superior longitudinal fasciculus. Damage to these pathways may be a useful biomarker for impaired informative spoken discourse and informs development of neurorehabilitation strategies.

## Teaser

Ding *et al*. report that the amount and speed of producing discourse-relevant content in post-stroke aphasia were associated with damage to anterior dorsal white matter pathways. Therefore, damage to these pathways may be a useful biomarker for impaired production of relevant information and informs development of neurorehabilitation strategies.

## Graphical Abstract

**Graphical Abstract:**
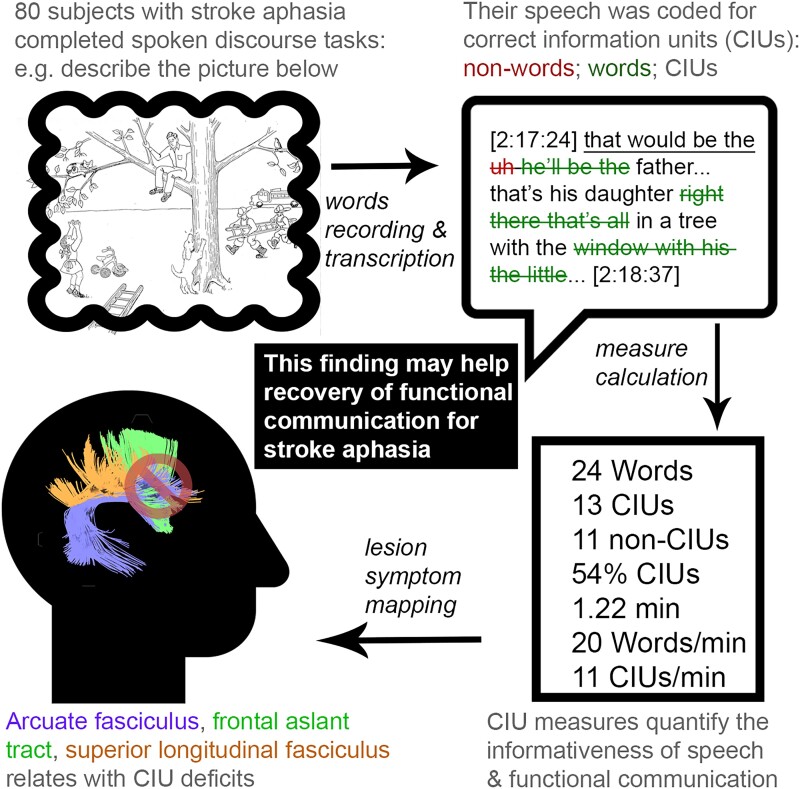

## Introduction

Aphasia is an impairment of language that occurs in up to 46% of patients in the acute phase after stroke, with about 60% of them still suffering from aphasia 1 year later.^1^ Post-stroke aphasia is associated with substantial negative effects on health and quality of life, including increased likelihood of death within 2 years of stroke, longer stays in hospital and reduced participation in activities across all domains of daily life.^2^ The ability to communicate a message in a real-world setting plays a key role in those activities, making it a critical aspect of recovery. However, current cognitive and neurocomputational models of language focus on subsystems (phonology, semantics, syntax, etc.) and do not address one’s ability to effectively and efficiently communicate an intended message.

Correct information unit (CIU) analysis is a well-established approach to quantifying the informativeness of spoken discourse by counting the relevant words, rate of production per unit of time and percentage of output. CIU analysis has standardized scoring rules, high inter-rater reliability, test–retest stability, differentiating sensitivity and ecological validity.^3^ Speech samples for CIU analysis can be elicited in different genres: descriptions of pictures, generic procedures/routines or personal information.^4^ Studies have found that these different genres produce differences in CIU measures,^5^ making it unclear whether the CIU scores derived from different elicitation genres are comparable. In addition, multiple CIU measures can be generated from a single sample: number of CIUs (#CIUs), CIUs per minute (CIUs/min) and CIUs per word (%CIUs). #CIUs reflects the quantity of information produced, while CIUs/min and %CIUs reflect the efficiency of information produced and informativeness produced, respectively.^5,6^ However, this intuitive distinction between quantity, efficiency and informativeness of spoken discourse may not be detectable if the measures are very highly correlated. This measurement problem may be especially difficult in relatively severe aphasia if all three aspects of spoken discourse are strongly impaired simultaneously.

Impaired CIU production may simply reflect general aphasia severity and/or total lesion size, as some studies have found.^6,7^ Beyond this general effect, tract-based analyses have found specific contributions of damage to the arcuate fasciculus (AF),^7-9^ a core component of the dorsal speech production system,^10^ and not of damage to grey matter regions or ventral tracts. However, a voxel-based lesion–symptom mapping (LSM) analysis using a similar measure (i.e. content units: generated based on a neurotypical cohort’s content rather than standardized rules) found a different pattern of results: no effect of total lesion size, dorsal white matter damage was associated with number of content units, but the content unit percentage was associated with damage to frontoparietal grey matter regions.^11^ Therefore, it remains unclear whether there are specific critical regions within the left hemisphere language system for communication and, if there are such specific regions, whether they are white matter tracts or grey matter processing hubs.

In the current study, we collected CIU scores from a large and unselected sample of participants with post-stroke aphasia. Relationships among different CIU measures (i.e. #CIUs, CIUs/min and %CIUs) derived from speech samples elicited with different prompts (i.e. simple question, complex picture and picture sequence) were examined to define appropriate composite measures. We then used three complementary LSM methods to identify regions that are critical for the production of CIUs beyond a general effect of total lesion size.

## Materials and methods

### Participants

The data set consisted of behavioural data from 80 participants who had suffered a left hemisphere stroke that produced some degree of language impairment. All participants were native English speakers, right-handed and able to produce some intelligible speech (i.e. name at least one picture on a picture naming test) without other severe disorders [i.e. dementia or neurodegenerative diseases, major psychosis, other nervous system insults (tumour, encephalitis, etc.), significant sensory disturbances (blindness in both eyes or profound deafness), developmental disability or seizures predating the stroke]. These participants were the subset who completed the spoken discourse elicitation task from a larger study of post-stroke aphasia without selection based on aphasia severity or type.^12^ Demographic and neuropsychological information is provided in Table 1. Western Aphasia Battery (WAB), a comprehensive and reliable aphasia evaluation for acquired neurological disorders, was conducted to evaluate participants’ language ability.^13,14^ The aphasia quotient was calculated based on the subtests of spontaneous speech, naming, comprehension and repetition and was used as a measure of aphasia severity. Aphasia subtypes were further classified according to standard WAB protocol based on the specific scores of those four subtests. Other widely used language tasks were also completed to measure naming, comprehension and repetition abilities: Philadelphia naming and word repetition tests, camel and cactus test and a non-word repetition test.^15,16^ The data collection and sharing were approved by the Institutional Review Board at the Einstein Healthcare Network; the neuroimaging was conducted at the University of Pennsylvania School of Medicine and approved by its Institutional Review Board. The analyses of de-identified data in the current study were approved by the University of Edinburgh PPLS Research Ethics Committee.

**Table 1:** Demographic and neuropsychological information

Variable	Num or mean, median	IQR	Range
Sex (Female:Male)	Num = 37:42		
Race (African-American:Caucasian)	Num = 37:42		
Age (years)	M = 59, Med = 59	51–68	31–79
Months post onset	M = 53, Med = 23	8–78	4–266
Education (years)	M = 15, Med = 14	12–18	10–21
Total lesion size (cc)	M = 99, Med = 77	48–133	5–376
WAB AQ (max 100)	M = 79, Med = 82	71–90	47–99
WAB fluency (max 10)	M = 7, Med = 8	5–9	2–10
WAB repetition (max 10)	M = 8, Med = 8	7–9	2–10
WAB comprehension (max 10)	M = 8, Med = 9	8–10	6–10
Non-word repetition (% correct)	M = 50, Med = 51	32–70	0–95
Camel and cactus test (% correct)	M = 76, Med = 80	72–83	31–94
Philadelphia naming test (% correct)	M = 73, Med = 79	15–97	64–86
Philadelphia repetition test (% correct)	M = 89, Med = 94	86–97	19–100
Number of words	M = 97, Med = 86	58–125	11–264
Number of CIUs	M = 56, Med = 54	28–74	6–161
CIUs/min	M = 47, Med = 43	20–66	7–137
%CIUs	M = 58%, Med = 60%	49–70%	25–86%
Apraxia of speech (severe:moderate:mild:none)	Num = 4:10:7:50		
Aphasia severity (severe:moderate:mild:recovered)	Num = 3:26:44:6		
Aphasia classification	Anomic: 43 Broca:18 Conduction: 10 Transcortical sensory: 2 Transcortical motor: 4 Wernicke: 2		

One participant did not provide their demographic information for research purposes, so the demographic data are for 79 participants. Ethnicity is not included in this table because no participants identified as Hispanic. CIU measures were averaged across all the tasks. CIUs, correct information units; WAB, Western Aphasia Battery. Aphasia severity categories are based on standard WAB AQ thresholds: severe, 26–50; moderate, 51–75; mild, >75; and recovered, >93.8.

### CIU assessment

#### Tasks

Spoken discourse samples were elicited in three genres (personal and procedural descriptions, complex picture descriptions and picture sequence descriptions) with multiple prompts in each genre.^3^ For the personal and procedural information genre, participants were asked to describe ‘what you usually do on Sundays’, ‘where you live and describe it’, ‘how to write and send a letter’ and ‘how to do dishes’. For picture description, participants were asked to look at a single complex picture and tell its story. This was repeated for four pictures: ‘cat in tree’, ‘birthday cake’,^3^ ‘cookie theft’^17^ and ‘picnic scene’.^13^ For picture sequence description, participants were presented with a sequence of pictures (‘farmer and his directions’ and ‘argument’)^3^ and asked to describe the depicted event(s). When participants’ speech was limited, they were encouraged to speak more but were not given any other prompts. Fourteen participants were missing up to five discourse samples (1 missing sample: 4; 2 samples: 2; 3 samples: 3; 4 samples: 4; 5 samples: 1).

#### CIU measures

The spoken discourse samples were audio recorded and transcribed with timing markers by four trained technicians, who then completed CIU scoring. There was no minimum number of elicited words required for data analysis, but each participant produced at least 108 words in total. A CIU was defined as a word that is intelligible, accurate, relevant and informative to the eliciting stimulus following the rules of Nicholas and Brookshire^3^ and a set of clarifications for cases not covered by those rules (https://osf.io/sudbt). For personal information, we scored CIUs based on words’ accuracy, relevance and informativeness to the topic, irrespective of its accuracy in the participant’s life. Each rater always evaluated a participant’s entire set of samples. Raters were thoroughly trained on the CIU scoring protocol. This study combines data from two bouts of data collection. In the first bout, 23 samples were scored by three raters. All three raters scored six participants’ samples to test inter-rater reliability. The overall mean agreement for words was 98% (pairwise agreement 98–100%) and for CIUs was 95% (pairwise agreement 94–96%). All the genres had excellent reliability (personal/procedural information: words 97% and CIUs 96%; complex picture: words 100% and CIUs 95%; and sequence pictures: words 99% and CIUs 93%). In the second bout, 57 participants’ samples were scored by a single rater. Forty-five samples from five participants were scored twice by the fourth rater to test intra-rater reliability. Overall, there was high reliability of words (agreement = 96%) and CIUs (agreement = 94%). All the specific genres reached excellent reliability (personal/procedural information: words 95% and CIUs 95%; complex picture: words 95% and CIUs 95%; sequence pictures: words 97% and CIUs 92%). Three standard CIU measures were calculated: #CIUs, CIUs/min and CIUs per cent (#CIUs/number of intelligible words) (Table 2).

**Table 2:** The median and interquartile values of CIU measures by tasks

Tasks	#CIUs	CIUs/min	%CIUs
Personal/procedural information	Med = 45, 21–70	Med = 43, 24–63	Med = 62, 49–73
What you do on Sundays	Med = 48, 27–91	Med = 51, 29–73	Med = 65, 53–80
How to wash dishes	Med = 34, 18–58	Med = 39, 17–65	Med = 56, 39–69
How to write letters	Med = 39, 17–63	Med = 42, 25–67	Med = 63, 48–75
Where you live	Med = 47, 24–83	Med = 43, 24–57	Med = 67, 45–78
Complex picture	Med = 55, 27–74	Med = 43, 21–68	Med = 61, 47–69
Cat in tree	Med = 50, 30–77	Med = 43, 22–75	Med = 66, 46–74
Cookie theft	Med = 41, 26–59	Med = 41, 18–62	Med = 59, 46–72
Birthday cake	Med = 50, 27–84	Med = 48, 18–77	Med = 60, 41–70
Picnic scene	Med = 53, 30–86	Med = 39, 21–57	Med = 61, 49–69
Sequence pictures	Med = 58, 29–87	Med = 42, 25–65	Med = 59, 45–70
Farmer and his directions	Med = 60, 30–93	Med = 37, 26–59	Med = 56, 45–69
Family argument	Med = 53, 30–88	Med = 44, 25–70	Med = 62, 48–74

CIU, correct information units; Med, median.

### Lesion data

Lesion masks were available for 66 (48 MRI and 18 CT) of the 80 participants. The masks were manually drawn according to an established protocol^18^ as in our prior work.^19^ For the MRI scans, lesions were manually segmented on each participant’s T_1_-weighted structural image by a trained technician and reviewed by an experienced neurologist for accuracy. Each participant’s brain image was registered to the Montreal Neurological Institute space Colin27 template by an automated symmetric diffeomorphic registration algorithm,^20^ and this image transformation solution was then applied to the lesion mask to register it to the same template. For the CT scans, an experienced neurologist drew the lesions directly onto the Colin27 template after rotating it (pitch only) to match the approximate slice plane of the participant’s scan.

### Statistical analyses

#### Behavioural analysis

First, correlations of CIU measures between 10 discourse tasks were performed to investigate the genre consistency rather than the genre difference. Correlation coefficients between genres and within genres were further compared by a two-sample *t*-test to examine whether consistency was higher within genre than between genres. Then, correlations between averaged CIU measures (#CIUs, %CIUs and CIUs/min) were calculated to assess their consistency. A principal component analysis (PCA) was used to assess the relationships between CIU measures and other measures of language ability. Horn’s parallel analysis was used to determine the component number retained. A varimax rotation was adopted to facilitate interpretation of components. Finally, we also examined the correlations between CIU measures and total lesion size, a common proxy for severity of neurological damage.

#### Lesion LSM

To investigate the lesion correlates of CIU impairment, we performed a multivariate voxel-wise LSM using the SCCAN algorithm implemented in the R package LESYMAP.^21^ We averaged CIU scores across 10 speech samples as the dependent variable. Total lesion size was regressed out from each CIU measure before LSM. Only voxels where at least 10% of the participants had damage were included. SCCAN is an optimization algorithm that finds an optimally ‘sparse’ set of weights that maximize the relationship between behavioural scores and voxel lesion values, i.e. a small set of voxels where lesions predict the behavioural measure effectively. The multivariate solution was evaluated by 4-fold cross-validation (CV) using correlation between expected and actual CIU measures. If a model was statistically significant (*P* < 0.05), we display voxels with normalized weights > 0.1, because they are most influential to predicting symptom severity. These maps are also described with reference to the AAL3 grey matter atlas regions^22^ and HCP1065 white matter atlas regions.^23^

#### Disconnection LSM

Voxel-wise disconnection LSM is a new approach to investigate lesion–symptom associations that result from structural white matter damage, even for regions that are spared after stroke.^24,25^ Disconnection maps were generated using the ‘lesion quantification toolkit’.^26^ Voxel-wise disconnection percentage was calculated by dividing each voxel’s number of disconnected streamlines by the total number of streamlines that pass through it in the template. These disconnection percentages were binarized at a threshold of 50% disconnection percentage.^24^ This approach was selected for better consistency with the voxel-wise lesion LSM, which is based on binary voxel lesion status (lesioned versus not lesioned). Multivariate SCCAN disconnection LSM was performed on CIU measures, analogous to the LSM analyses described above (>10% of participants; controlling for total lesion size).

#### Tract-based LSM

We performed a tract-based LSM to validate findings from the previous literature and our voxel-wise analyses. Complementary to the voxel-wise analyses, this analysis considers white matter tracts to function as a whole, so the symptom severity is dependent on amount of damage to the tract, irrespective of which part of the tract may be damaged. Language-relevant white matter tracts were selected for analysis:^27^ AF, frontal aslant tract (FAT), inferior fronto-occipital fasciculus, inferior longitudinal fasciculus, the three parts of the superior longitudinal fasciculus (SLF1–3) and uncinate fasciculus. Lesion percentage was computed for each tract by overlaying lesions on those tracts extracted from the HCP1065 atlas. Then, partial correlations between CIU measures and each tract’s lesion percentage were calculated, accounting for the effect of total lesion size.

#### Validation analysis

Several validation analyses were conducted to confirm our results were not affected by potential confounding variables. First, aphasia type is a crucial factor that may differentially affect performance across spoken discourse genres. We split participants according to three major aphasia types: anomic/mild (*n* = 43), fluent (Wernicke’s, conduction and transcortical sensory; *n* = 14), and non-fluent (global, Broca’s and transcortical motor; *n* = 22).^28^ Then, we re-calculated the genre consistency analyses in each group. Second, it is possible that some effects were driven by a small subset of especially severely impaired individuals. We removed participants who produced less than 20 CIUs (*n* = 13) and re-ran the LSM analyses. Third, to mitigate the effects of motor speech disorders on the LSM results, we further controlled for non-word repetition performance and apraxia of speech in the LSM analyses. Fourth, to assess how genre differences affected the LSM analyses, we also ran LSM analyses separately for each genre (i.e. personal/procedural information, complex picture and picture sequence). Fifth, the main analyses used the Colin27 template, which is a young adult brain. The Colin27 is a standard and widely used template; however, most of the participants in this study were older adults (typical of the stroke survivor population), and there are notable brain changes in aging, such as white matter hyperintensity.^29^ To avoid age-related bias, we further registered the young adult brain template to the older adult MIITRA template.^30^ The warping information was used to transform all the lesion masks, and main analyses were re-done using the updated masks. Finally, because of the high correlation between CIU measures and the WAB fluency measure, we provided the LSM results of WAB fluency scores.

## Results

### Behavioural results

**Figure 1:**
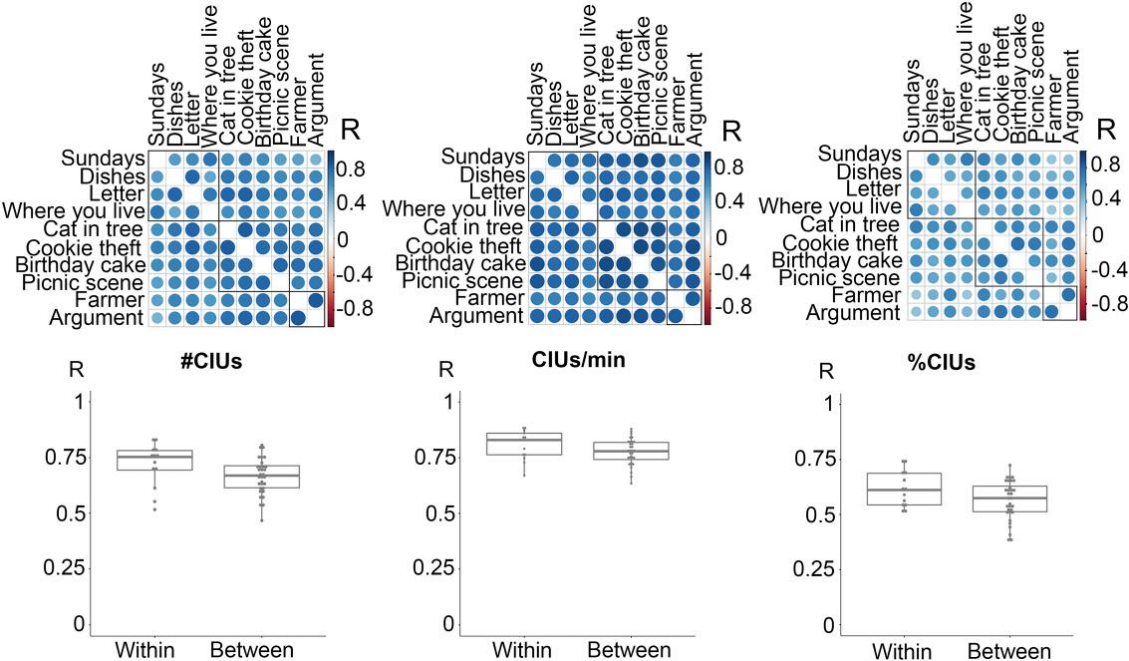
**Correlation coefficients within and across elicitation genres.** Elicitation genres include personal and procedural information, complex picture description and picture sequence description (outlined by three squares under the correlation matrix). First row shows Pearson correlation matrices (*n* = 80). Each data cell represents a correlation coefficient for pairs of prompts. The box plots indicate medians, interquartile range and range. Each data point represents a correlation coefficient for pairs of prompts within or between genres (*n* = 45). Welch two sample *t*-tests were conducted, and none of the comparisons were significant (*t* < 1.96, *P* > 0.06).

CIU measures had high consistency across all prompts and genres (*r* > 0.38, *P* < 0.0009; Fig. 1). Correlations between CIU measures based on prompts from the same genre were not statistically significantly higher than correlations between prompts from different genres (*t* < 1.96; *P* > 0.06; see Fig. 1). These results indicate that CIU measures are highly consistent across elicitation genres.

#CIUs and CIUs/min were strongly correlated (*r* = 0.73, *P* < 0.0001), and they were moderately correlated with %CIUs (*r* > 0.44, *P* < 0.0001; see Fig. 2), which suggests different CIU measures were only weakly dissociable in post-stroke aphasia. A PCA was used to examine the relationship between the CIU measures and other neuropsychological and language tests (Table 3). Based on a parallel analysis, three components were extracted: fluency, phonology and semantics. These three components explained 60–80% of variance in the CIU measures. The CIU measures were most strongly related with the fluency component (loadings > 0.63), though %CIUs was additionally related to phonology (loading = 0.24) and semantics (loading = 0.39). The loading of %CIUs on the semantic component was relatively unique among CIU measures, suggesting a distinctive role of semantic ability in %CIUs relative to its role in #CIUs and CIUs/min. All CIU measures were also significantly correlated with total lesion size (*r* < −0.29, *P* < 0.02), indicating that overall amount of damage within the left middle cerebral artery territory predicts quantity, efficiency and informativeness of spoken discourse (see Fig. 2).

**Figure 2:**
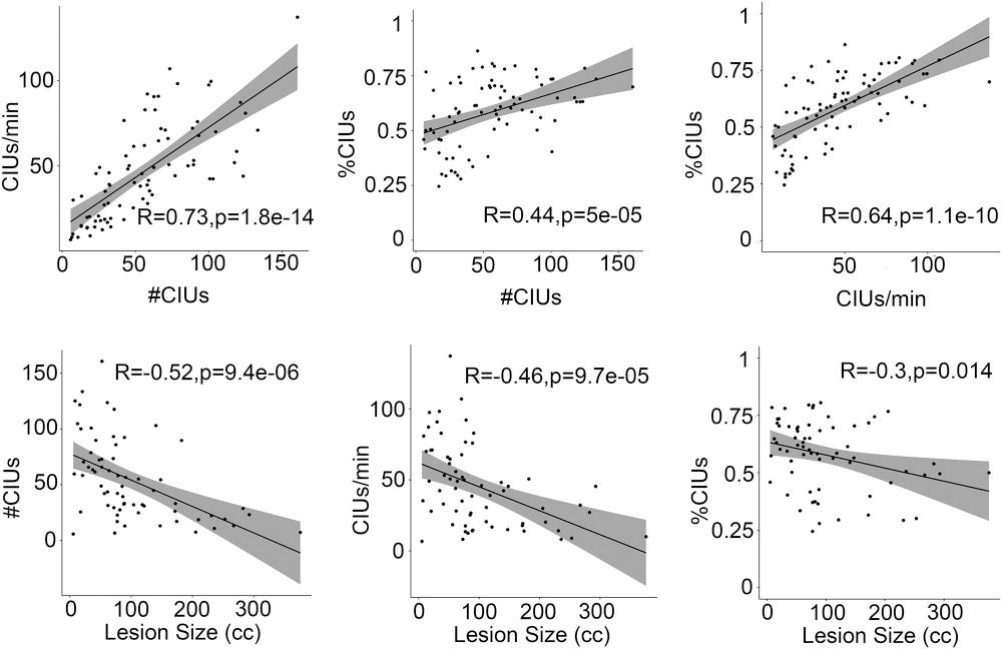
**Correlations between CIU measures and with total lesion size.** All the analyses here are Pearson correlations. Each data point represents each patient. *R*- and *P*-values are labelled in the figure. The shaded regions indicate 95% confidence intervals.

**Table 3:** PCA loadings and total variance explained

Measures	C1: Fluency	C2: Phonology	C3: Semantic	Variance
#CIUs	**0.85**	−0.04	0.03	73%
%CIUs	**0**.**63**	0.24	0.39	60%
CIUs/min	**0**.**86**	0.18	0.13	79%
WAB-AQ	**0**.**81**	0.38	0.35	92%
WAB fluency	**0**.**88**	0.18	0.10	82%
WAB comprehension	**0**.**52**	0.04	**0**.**62**	65%
WAB repetition	**0**.**58**	**0**.**54**	0.06	64%
Non-word repetition	0.26	**0**.**83**	0.00	75%
PNT: accuracy	0.34	**0**.**65**	**0**.**57**	86%
PNT: semantic errors	−0.17	0.14	**−0**.**78**	65%
PNT: phonological errors	−0.14	**−0**.**90**	0.10	83%
Semantic discrimination	0.10	0.31	**0**.**69**	58%
CCT	0.01	−0.18	**0**.**77**	62%
PRT	0.02	**0**.**92**	−0.01	85%

PCA loadings > 0.50 are in bold. AQ, aphasia coefficient; CCT, camel and cactus test; CIU, correct information unit; PCA, principal component analysis; PNT, Philadelphia naming test; PRT, Philadelphia repetition test; WAB, Western Aphasia Battery.

### LSM results

**Figure 3:**
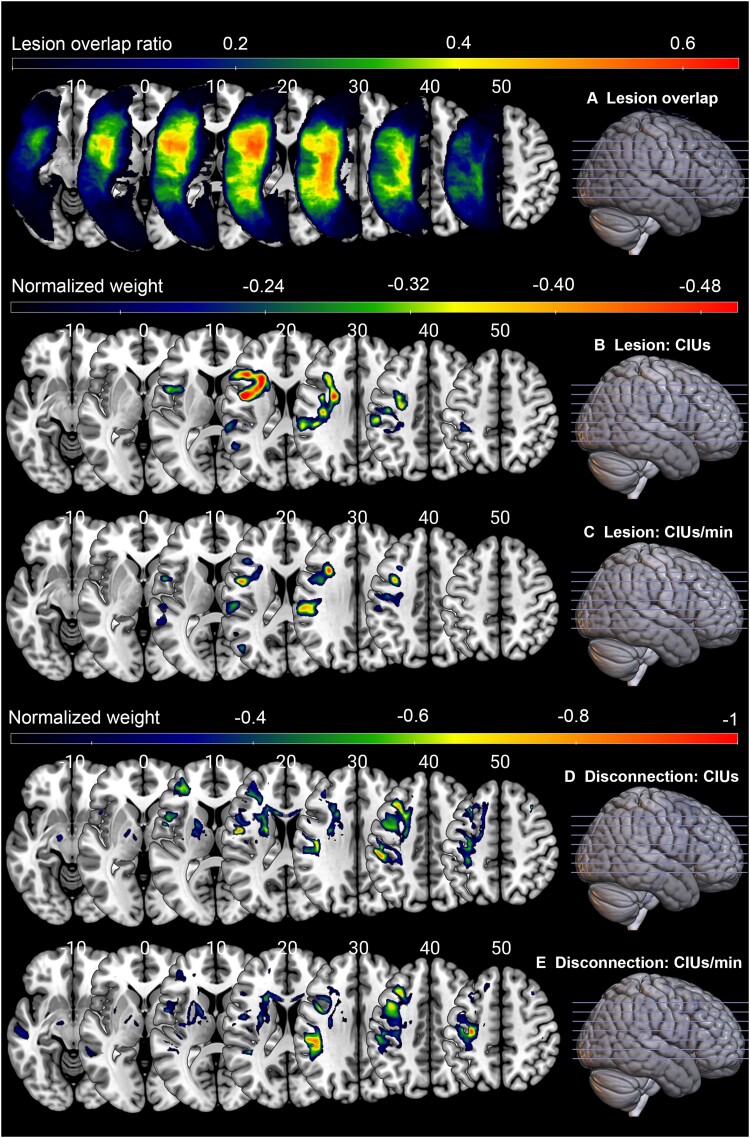
**Lesion correlates of CIU measures.** (**A**) Lesion overlay map (*n* = 66). (**B**) Lesion LSM solution for #CIUs. (**C**) Lesion LSM solution for CIUs/min. (**D**) Disconnection analysis result for #CIUs. (**E**) Disconnection analysis result for CIUs/min. The lesion and disconnection LSM used participants’ lesion maps and inferential disconnection map (binarized by disconnection proportion of 0.5), respectively to predict CIU measures. Total lesion size was regressed out from the symptom score. Values in the brain map correspond to standardized weights, indicating the contribution of each voxel to the model. Only voxels with normalized weights greater than 0.1 are shown. Warmer colours represent stronger contributions. LSM analyses for %CIUs did not produce a statistically significant solution, so they are not included here.

Lesion overlap is shown in Fig. 3A and reflects good coverage of the left MCA territory typical for post-stroke aphasia.

#### Lesion LSM

Lesion LSM produced a marginally significant solution for #CIUs (optimal sparseness = 0.70, CV correlation = 0.24, *P* = 0.05) and a statistically significant solution for CIUs/min (optimal sparseness = 0.49, CV correlation = 0.36, *P* = 0.003) but not for %CIUs (CV correlation = 0.15, *P* = 0.22). Figure 3B and C shows these solutions (only voxels with normalized weights above 0.1). Most of the voxels were located in anterior portions of dorsal stream white matter tracts. For #CIUs, these regions included the left AF, corpus callosum, FAT and supramarginal gyrus. For CIUs/min, the regions included the left AF, FAT, SLF2, supramarginal gyrus and precentral gyrus (Table 4).

**Table 4:** Summary of LSM results for #CIUs and CIUs/min (%CIUs not included because there were no statistically significant results)

	White matter	Grey matter
#CIUs		
Lesion LSM	AF 20%, FAT 18%, CC 13%	SMG 10%
Disconnection LSM	CC 20%, FAT 16%, SLF2 12%, TR_S 11%, CPT_F 10%, CST 10%	PreCG 13%, MFG 11%
Tract-wise LSM	AF −0.26*, FAT −0.39***, SLF2 −0.37**	
CIUs/min		
Lesion LSM	AF 18%, FAT 17%, SLF2 10%	SMG 16%, PreCG 13%
Disconnection LSM	CC 21%, SLF2 11%	PreCG 13%
Tract-wise LSM	AF −0.40***, FAT −0.35**, SLF2 −0.31**, SLF3 −0.34**	

Lesion/disconnection LSM presents the percentage of results map in atlas regions (only regions containing >10% of the result map are shown). The tract-wise LSM presents the partial correlation coefficients. All the regions are in the left hemisphere. *P < 0.05; **P < 0.01; ***P < 0.001. AF, arcuate fasciculus; CC, corpus callosum; CIU, correct information unit; CPT_F, frontal corticopontine tract; CST, corticospinal tract; FAT, frontal aslant tract; LSM, lesion symptom mapping; MFG, middle frontal gyrus; PreCG, precentral gyrus; SLF, superior longitudinal fasciculus; SMG, supramarginal gyrus; TR_S, superior thalamic radiation.

#### Disconnection LSM

The lesion LSM results indicated that white matter damage is the key contributor to the quantity and efficiency of spoken discourse (as measured by CIU analysis). To evaluate this further, we performed a disconnection LSM, which is a more sensitive approach to evaluating the effects of white matter disruption.^25^ The solution was significant for #CIUs (optimal sparseness = 0.50, CV correlation = 0.51, *P* = 0.00001) and CIUs/min (optimal sparseness = 0.66, CV correlation = 0.41, *P* = 0.0005). The solution for %CIUs was again not statistically significant (CV correlation = 0.11, *P* = 0.36). Figure 3D and E shows these solutions (only voxels with normalized weights above 0.1). Consistent with the lesion LSM findings, most solution-relevant voxels were in the dorsal white matter pathways. For #CIUs, the critical white matter tracts were left corpus callosum, FAT, SLF2, frontal corticopontine tract, corticospinal tract and superior thalamic radiations, and the grey matter regions were left precentral gyrus and middle frontal gyrus. For CIUs/min, the critical white matter tracts were left corpus callosum and SLF2, and there were also critical voxels in the left precentral gyrus (Table 4).

#### Tract-wise LSM

**Figure 4:**
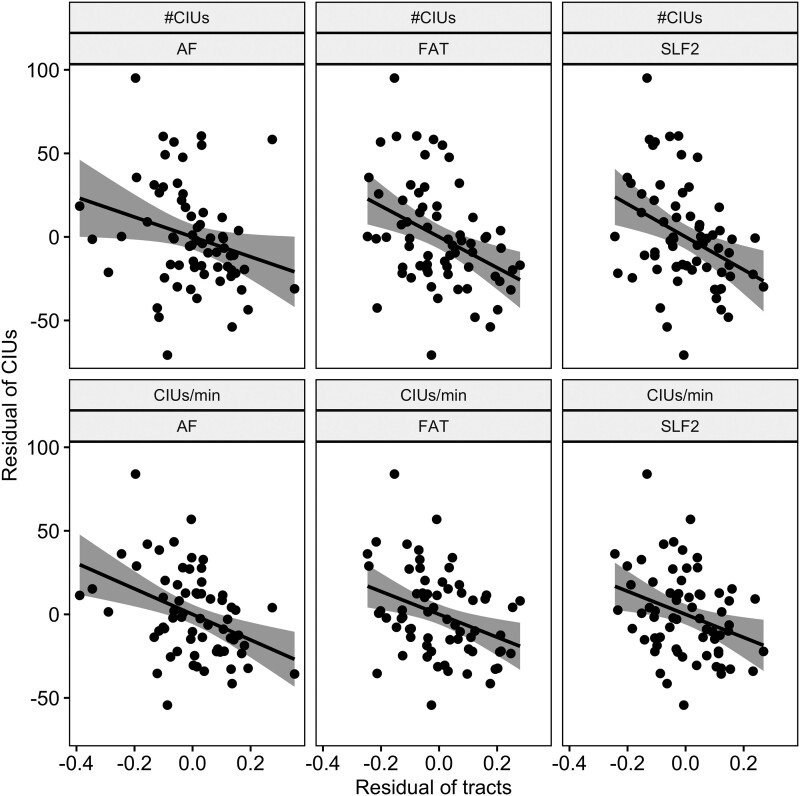
**Partial correlations between dorsal white matter tracts and #CIUs and CIUs/min.** The partial correlations were conducted between the damage of white matter tracts and CIU measures, controlling for total lesion size. Each data point represents a participant. The shaded regions are 95% confidence intervals. Only significant correlations are displayed here (*r* < −0.25, *P* < 0.02).

For both the lesion LSM and disconnection LSM analyses, the crucial voxels for CIU measures largely overlapped with dorsal white matter pathways, but only minimally with grey matter regions. As an additional validation of this finding, we performed a tract-wise analysis. As shown in Table 4 and Fig. 4, after controlling for total lesion size, #CIUs and CIUs/min significantly correlated with the dorsal pathways, including AF, FAT and SLF, but not with the ventral pathways (uncinate fasciculus, inferior fronto-occipital fasciculus or inferior longitudinal fasciculus). No tracts significantly correlated with %CIUs (all *P* > 0.05). In sum, all three sets of analyses converge to indicate that #CIUs and CIUs/min are associated with damage in dorsal white matter pathways, while there are no statistically significant lesion correlates for %CIUs.

#### Validation analyses

First, to investigate the interaction of aphasia subtype and CIU genre consistency, we separated participants into anomic (*n* = 43), fluent (*n* = 14) and non-fluent (*n* = 22) groups (Supplementary Table 1). Consistency was moderate or high in all groups for all CIU measures; however, ANOVA showed a significant interaction between aphasia subtypes and CIU measures (*F*(4,396) = 8.8, *P* < 0.001). Further *post hoc* tests found that, for #CIUs, the anomic and fluent groups had significantly higher consistency than the non-fluent group (difference > 0.12, *P* < 0.006). For %CIUs and CIUs/min, consistency was significantly higher in the anomic group than the fluent and non-fluent groups (difference > 0.10, *P* < 0.04).

To remove the influence of very severely impaired participants, we excluded 20 participants with CIUs < 20 in the LSM analysis. The results of disconnection and tract-wise LSM did not change compared with the original one, except that %CIUs became correlated with the AF damage (*r* = −0.32, *P* = 0.009). None of the lesion LSM models produced significant results for this smaller and more restricted sample (*r* < 0.24, *P* > 0.07) (Supplementary Table 2).

To mitigate the potential influence of motor function to the LSM, we added the non-word repetition task or apraxia of speech as a covariate. When controlling for the repetition, the effects of the dorsal white matter pathways remained, even for the measure of %CIUs (see Supplementary Table 3). Moreover, %CIUs showed significant correlation with posterior grey matter regions (e.g. superior and middle temporal gyri). The lesion LSM for #CIUs and CIUs/min did not find any significant correlates (*r* < 0.20, *P* > 0.11). For analyses with apraxia of speech as a covariate, the effects of the dorsal white matter pathways remained only for the disconnection and tract LSM but not the lesion LSM (*r* < 0.15, *P* > 0.24) (see Supplementary Table 4).

To examine the effect of genre on LSM, we ran LSM analyses separately for different elicitation genres. The results were basically similar across genres confirming the importance of dorsal white matter pathways (see Supplementary Table 5). Regarding the difference, the lesion LSM of #CIUs was not significant in the genres of personal information and single picture (*r* < 0.15, *P* > 0.24). For the genre of picture sequence, the lesion LSM of #CIUs found a correlation of inferior frontal gyrus. The tract LSM of %CIUs found a correlation of AF (*r* = −0.25, *P* = 0.02).

Because the young adult brain template may not be optimal for this older stroke survivor sample, we transformed the lesion masks to the MIITRA older adult brain template and re-ran the analyses. The dorsal white matter pathways remained in the lesion and tract-wise LSM, although not in the disconnection LSM (see Supplementary Table 6). The tract LSM showed a significant correlation between %CIUs and AF (*r* = −0.22, *P* = 0.04).

Because of the high correlation of WAB fluency and CIU measures, we examined the lesion correlates of WAB fluency. WAB fluency was more strongly associated with damage to the AF and temporal gyri and less strongly with the other dorsal white matter pathways and precentral gyrus (see Supplementary Table 7).

In sum, the results of these validation analyses are broadly consistent with the main analyses. Although there are small differences in these results, they do not change the core finding that anterior dorsal white matter damage is the critical lesion correlate of impaired production of informative speech.

## Discussion

The present study systematically explored the lesion correlates of informative speech in a large sample of participants with post-stroke aphasia. CIU measures were highly consistent across elicitation genres and had similar lesion correlates. Information quantity (#CIUs) and speed (CIUs/min) were highly correlated with each other and with total lesion size. Beyond lesion size, these measures were specifically associated with damage to dorsal white matter pathways, particularly the AF and FAT. Informativeness (%CIUs) was less strongly correlated with the other measures and was not reliably associated with damage to specific brain regions within the left hemisphere language network.

Spoken discourse can be elicited in different ways,^4,31^ sometimes leading to divergent linguistic measures because of factors such as genre type (e.g. narrative versus procedural),^32,33^ complexity (e.g. number of pictures) and presence or absence of cues (e.g. pictures).^34-36^ In the present sample of 80 participants with post-stroke aphasia, CIU measures derived from 10 different prompts in three different genres (procedural/personal, complex picture and picture sequence descriptions) displayed high convergence, both within genres and across genres. Therefore, the present results converge with previous findings that content measures from procedural, complex picture and personal descriptions statistically cluster together,^11^ indicating informative speech deficits can traverse genres. However, larger discrepancies may emerge for different linguistic measures (e.g. lexical diversity) or cohorts with different aphasia severity (e.g. less severe patients).^33,37-39^ These results indicate high consistency across genres, though using fewer elicitation samples may be risky because it is more difficult to reach high reliability for CIUs based on smaller speech samples (e.g. <300 words or 4 stimuli).^4^

It has been suggested that the different CIU measures represent different cognitive abilities: #CIUs for quantity of information, CIUs/min for efficiency of information and %CIUs for informativeness.^5,6^ In the present data, #CIUs and CIUs/min were highly correlated and were associated with the same lesion locations. These measures appear to be strongly related to fluency. A somewhat different pattern was observed for %CIUs, which was less strongly correlated with the other measures and not significantly related with any brain regions, though it was somewhat associated with semantic measures, possibly suggesting a stronger link to lexical-semantic access deficits. These results suggest that the quantity and efficiency of information produced in post-stroke aphasia appear to be only weakly dissociable, while informativeness itself is more distinct.

Total lesion size is a good predictor of aphasia severity and other high-level language functions,^40,41^ including length and complexity of sentences.^42^ However, prior studies reported mixed results regarding whether information content is^7^ or is not^11^ associated with lesion size. In the present data, all three CIU measures were highly correlated with total lesion size. The present sample was substantially larger than those previous studies (*n* = 66, compared with *n* = 30 and *n* = 46), so the current state of the evidence supports an association between total lesion size and spoken discourse production deficits. Because producing relevant information requires coordinating neural subsystems that support different language and cognitive functions, it is plausible that overall lesion size—rather than damage to any particular brain region(s)—is the best predictor of impaired information production. This plausible causal relationship between total lesion size and CIU impairment means that identifying specific lesion correlates (i.e. LSM) requires controlling for lesion size.^43,44^

In the present study, three sets of converging analyses found that, after controlling for lesion size, impaired quantity (#CIUs) and efficiency (CIUs/min) of relevant information in spoken discourse were associated with damage to dorsal white matter pathways: the AF, FAT and SLF. This converges with prior evidence that AF damage is associated with informative speech impairment and its recovery^7,9,11,45^ and that FAT^11,46,47^ and SLF3 (i.e. anterior AF)^11,46,48^ damage are associated with speech fluency and informative discourse and SLF2’s involvement in language motor planning.^49^ The present study used the largest CIU sample to date and three state-of-the-art LSM analysis methods. Together, they provide strong and convergent evidence that producing relevant information efficiently in spoken discourse critically and specifically relies on anterior dorsal stream white matter tracts. We also found that number and speed of CIUs were related with several grey matter regions, including the precentral, supramarginal and middle frontal gyri, even though the pattern was inconsistent across different LSM methodologies. This difference likely reflects key differences between the methodologies. Lesion LSM identifies regions where damage predicts symptom severity, while disconnection LSM identifies regions where connectivity is associated with the symptom. It may be that, for grey matter regions, #CIUs and CIUs/min are associated with damage to supramarginal gyrus and with disconnection of precentral gyrus. Regarding informativeness (%CIUs), we did not find any significant associations in the brain. This may be because informativeness is more related to semantics, which relies on bilateral brain regions.^50,51^ The current data did not include any participants with damage in the right hemisphere, so we are not able to test this speculation.

Besides the total lesion size, other potentially confounding variables also slightly influenced the results. Controlling for other variables tended to decrease the statistical significance of lesion LSM models but had less impact on disconnection LSM. This is likely because disconnection LSM has higher sensitivity,^25,52^ especially when—as we argue—the symptoms under investigation are more strongly associated with disconnections than with local damage. A surprising effect was that controlling for repetition led to statistically significant effects for %CIUs in posterior grey matter areas, such as superior and middle temporal gyri. Damage in these areas is associated with performance in semantic comprehension tasks,^53,54^ suggesting that regressing out motor speech deficits made the effect of semantic deficits easier to find. Specifically for the picture sequence description genre, #CIUs was associated with damage in the inferior frontal gyrus, which is associated with semantic cognition, particularly semantic control.^55-57^ It may be that semantic control is particularly important for producing extensive relevant information (#CIUs) during picture sequence description. Changing to the older adult template, which should better fit the stroke cohort, only affected the results of disconnection LSM, suggesting that disconnection LSM is more sensitive to differences between templates. Finally, WAB fluency correlated with CIU measures behaviourally and showed similar LSM results, such as the AF and supramarginal gyrus. However, #CIUs and CIUs/min were more strongly associated with damage to other dorsal white matter pathways and frontal regions, while WAB fluency was more strongly associated with damage to temporal gyri. WAB fluency is widely used because it is part of a standard aphasia assessment, but it is assigned with reference to qualitative descriptions of mixed cognitive subcomponents, including articulation, grammar and semantics.^19,58^ This makes it a psychometrically and cognitively problematic measure. Studying the neural basis of CIUs could help unpack the nebulous label of ‘fluency’ and lead to a better understanding of functional communication.

Improvement in functional communication is a major therapy outcome desired by people with aphasia and their caregivers.^59,60^ Designed as a measure for this aim, CIUs provide an opportunity to accurately quantify the deficits instead of coarse qualitative judgements. CIU analysis showed good psychometric properties: high convergence and reliability in CIU measures within and across genres suggest clinicians may be able to assess potential treatment effects on discourse production with various elicitation topics. Also, mapping out the neural substrate for discourse production could inform future studies that seek to identify the neural bases of generalization to discourse production from microlinguistic, impairment-based treatment approaches.

The data used in the current study were limited in several ways. First, the present results shed light on persistent impairments in spoken discourse but not on recovery of spoken discourse production. Which brain regions are critical for recovery of informative spoken discourse remains to be revealed by appropriate longitudinal studies.^61^ Second, the present analyses focused on the quantity and efficiency of producing discourse-relevant information but not on alternative communication strategies. Such strategies used by individuals with aphasia (e.g. naming objects while sacrificing grammatical structure) deserve further research. Third, we did not find any lesion correlates for discourse informativeness, which may rely on brain regions bilaterally. Future studies will need to include participants with right hemisphere damage to test this hypothesis.

In sum, this study revealed that, in chronic post-stroke aphasia, production of relevant information in spoken discourse was highly correlated across elicitation genres and prompts. Most importantly, impairments in the quantity and speed of informative speech production (#CIUs and CIUs/min) were associated with damage to anterior dorsal white matter pathways, indicating that these are critical neural substrates and possible biomarkers for impaired informative communication.

## Supplementary Material

fcad310_Supplementary_Data

## Data Availability

The data are available on our OSF project page (https://osf.io/su9ka/).
