# Supplementary material for: Impaired discourse content in aphasia is associated with frontal white matter damage
Source: Brain Commun. 2023 Nov 10;5(6):fcad310. doi: 10.1093/braincomms/fcad310 (PMC10664411; doi:10.1093/braincomms/fcad310)
Supplement: fcad310_Supplementary_Data [file fcad310_supplementary_data.docx]

**Supplementary Table 1. The median and interquartile values of CIU genre consistency (correlation coefficients) across aphasia subtypes**

|  | Total r value | Within genre r | Between genre r |
| --- | --- | --- | --- |
| #CIUs |  |  |  |
| Anomic | 0.59 (0.52-0.67) | 0.62 (0.57-0.72) | 0.55 (0.51-0.66) |
| Fluent | 0.61 (0.53-0.74) | 0.72 (0.56-0.76) | 0.60 (0.52-0.67) |
| Nonfluent | 0.55 (0.28-0.69) | 0.60 (0.55-0.75) | 0.46 (0.26-0.64)* |
| CIUs/min |  |  |  |
| Anomic | 0.73 (0.67-0.78) | 0.74 (0.70-0.80) | 0.71 (0.65-0.76) |
| Fluent | 0.50 (0.24-0.62) | 0.50 (0.23-0.61) | 0.47 (0.28-0.62) |
| Nonfluent | 0.40 (0.25-0.55) | 0.47 (0.37-0.61) | 0.35 (0.24-0.50) |
| %CIUs |  |  |  |
| Anomic | 0.56 (0.49-0.61) | 0.60 (0.39-0.66) | 0.55 (0.49-0.61) |
| Fluent | 0.46 (0.33-0.63) | 0.45 (0.39-0.65) | 0.48 (0.29-0.62) |
| Nonfluent | 0.46 (0.34-0.59) | 0.42 (0.34-0.73) | 0.46 (0.34-0.53) |

*: P < 0.05 when comparing within and between genre r values.

**Supplementary Table 2. Summary of LSM results without patients whose #CIUs was < 20**

|  | White matter | Grey matter |
| --- | --- | --- |
| #CIUs |  |  |
| Disconnection LSM | CC 20%, FAT 19%, SLF2 16% | PreCG 22%, MFG 14% |
| Tract-wise LSM | AF -0.28*, FAT -0.46***, SLF2 -0.49*** |  |
| CIUs/min |  |  |
| Disconnection LSM | AF 11%, CC 21%, SLF2 13% | PreCG 25%, PoCG 11% |
| Tract-wise LSM | AF -0.47***, FAT -0.40**, SLF2 -0.40**, SLF3 -0.30* |  |
| %CIUs |  |  |
| Tract-wise LSM | AF -0.32** |  |

Disconnection LSM presents the percentage of results map in atlas regions (only regions containing >10% of the result map are shown). The tract-wise LSM presents the partial correlation coefficients. All regions are in the left hemisphere. **p* < 0.05; ***p* < 0.01; ****p* < 0.001. LSM: lesion symptom mapping; CIU: correct information unit; AF: arcuate fasciculus; FAT: frontal aslant tract; CC: corpus callosum; SLF: superior longitudinal fasciculus; PreCG: precentral gyrus; PoCG: postcentral gyrus; MFG: middle frontal gyrus.

**Supplementary Table 3. Summary of LSM results controlling total lesion size and nonword repetition.**

|  | White matter | Grey matter |
| --- | --- | --- |
| #CIUs |  |  |
| Disconnection LSM | AF 10%, CC 21%, CST 12%, FAT16%, SLF2 13%, TR_S 11% | PreCG 14% |
| Tract-wise LSM | AF -0.28*, FAT -0.39***, SLF2 -0.33** |  |
| CIUs/min |  |  |
| Disconnection LSM | AF 12%, CC 27%, FAT 16%, TR_S 11% | PreCG 14% |
| Tract-wise LSM | AF -0.42***, FAT -0.41***, SLF2 -0.27*, SLF3 -0.25* |  |
| %CIUs |  |  |
| Lesion LSM | AF 14%, FAT 11%, SLF2 15% | PreCG 12%, ROL 15%, STG 33% |
| Disconnection LSM | CC 16% | PreCG 23%, MFG 14%, PCL 12%, MTG 20% |

Lesion/Disconnection LSM presents the percentage of results map in atlas regions (only regions containing >10% of the result map are shown). The tract-wise LSM presents the partial correlation coefficients. All the regions are in the left hemisphere. **p* < 0.05; ***p* < 0.01; ****p* < 0.001. LSM: lesion symptom mapping; CIU: correct information unit; AF: arcuate fasciculus; FAT: frontal aslant tract; CC: corpus callosum; SLF: superior longitudinal fasciculus; TR_S: superior thalamic radiation; CST: corticospinal tract; PreCG: precentral gyrus; MFG: middle frontal gyrus; ROL: Rolandic operculum; STG: superior temporal gyrus; PCL: paracentral lobule; MTG: middle temporal gyrus.

**Supplementary Table 4. Summary of LSM results controlling total lesion size and apraxia of speech.**

|  | White matter | Grey matter |
| --- | --- | --- |
| #CIUs |  |  |
| Disconnection LSM | AF 11%, CC 19%, CPT_F 16%, CST 13%, FAT19%, TR_S 14% | PreCG 10%, MFG 15% |
| Tract-wise LSM | AF -0.21*, FAT -0.33**, SLF2 -0.29* |  |
| CIUs/min |  |  |
| Disconnection LSM | AF 10%, CC 20%, SLF2 12% | PreCG 37%, MFG 14% |
| Tract-wise LSM | AF -0.36**, FAT -0.28*, SLF3 -0.23* |  |

Disconnection LSM presents the percentage of results map in atlas regions (only regions containing >10% of the result map are shown). The tract-wise LSM presents the partial correlation coefficients. All regions are in the left hemisphere. **p* < 0.05; ***p* < 0.01; ****p* < 0.001. LSM: lesion symptom mapping; CIU: correct information unit; AF: arcuate fasciculus; FAT: frontal aslant tract; CC: corpus callosum; CPT_F: frontal corticopontine tract; CST: corticospinal tract; SLF: superior longitudinal fasciculus; PreCG: precentral gyrus; MFG: middle frontal gyrus.

**Supplementary Table 5. Summary of LSM results using different genres**

|  | White matter | Grey matter |
| --- | --- | --- |
| Personal/Procedural information |  |  |
| #CIUs |  |  |
| Disconnection LSM | CC 19%, CPT_F 13%, CST 13%, FAT 17%, SLF2 12%, TR_S 12% | MFG 11% |
| Tract-wise LSM | AF -0.25*, FAT, -0.35**, SLF2 -0.31** |  |
| CIUs/min |  |  |
| Lesion LSM | AF 15%, FAT 13%, SLF2 10%, SLF3 11% | PreCG 13%, SMG: 22%, STG: 14% |
| Disconnection LSM | AF 11%, CC 21%, FAT 12% | PreCG 12% |
| Tract-wise LSM | AF -0.39***, FAT -0.34**, SLF2 -0.30**, SLF3 -0.39*** |  |
| Single picture |  |  |
| #CIUs |  |  |
| Disconnection LSM | CC 19%, CPT_F 12%, CST 11%, FAT 18%, SLF2 11%, TR_S 13% | PreCG 12%, MFG 12% |
| Tract-wise LSM | AF -0.22*, FAT, -0.36**, SLF2 -0.39*** |  |
| CIUs/min |  |  |
| Lesion LSM | AF 17%, FAT 14%, SLF2 11% | PreCG 11%, SMG 12%, MTG 14% |
| Disconnection LSM | AF 14%, CC 21% | PreCG 11% |
| Tract-wise LSM | AF -0.41***, FAT -0.32**, SLF2 -0.30**, SLF3 -0.31** |  |
| Picture sequence |  |  |
| #CIUs |  |  |
| Lesion LSM | AF 28%, CC 10%, FAT 17% | IFGtri 17% |
| Disconnection LSM | CC 20%, SLF2 10% | MFG 12%, IPL 11% |
| Tract-wise LSM | AF -0.27*, FAT, -0.36**, SLF2 -0.32** |  |
| CIUs/min |  |  |
| Disconnection LSM | CC 25%, FAT 12%, SLF2 10% | PreCG 12% |
| Tract-wise LSM | AF -0.33**, FAT -0.37**, SLF2 -0.31**, SLF3 -0.24* |  |
| %CIUs |  |  |
| Tract-wise LSM | AF -0.25* |  |

Lesion/Disconnection LSM presents the percentage of results map in atlas regions (only regions containing >10% of the result map are shown). The tract-wise LSM presents the partial correlation coefficients. All the regions are in the left hemisphere. **p* < 0.05; ***p* < 0.01; ****p* < 0.001. LSM: lesion symptom mapping; CIU: correct information unit; AF: arcuate fasciculus; FAT: frontal aslant tract; CC: corpus callosum; SLF: superior longitudinal fasciculus; TR_S: superior thalamic radiation; CPT_F: frontal corticopontine tract; CST: corticospinal tract; PreCG: precentral gyrus; MFG: middle frontal gyrus; SMG: supramarginal gyrus; MTG: middle temporal gyrus; IFGtri: inferior frontal gyrus, Pars triangularis; IPL: inferior parietal lobe.

**Supplementary Table 6. Summary of LSM results on the older adult template**

|  | White matter | Grey matter |
| --- | --- | --- |
| #CIUs |  |  |
| Lesion LSM | AF 23%, CC 18%, FAT 23%, SLF2 11%, TR_S 11% | SMG 10% |
| Disconnection LSM | CC 27% |  |
| Tract LSM | AF -0.24*, FAT -0.36**, SLF2 -0.30** |  |
| CIUs/min |  |  |
| Lesion LSM | AF 31%, FAT 25%, SLF2 22%, SLF3 10% | PreCG 31% |
| Disconnection LSM | CC 25% |  |
| Tract LSM | AF -0.42***, FAT -0.36**, SLF2 -0.27*, SLF3 -0.28* |  |
| %CIUs |  |  |
| Tract LSM | AF -0.22* |  |

Lesion/Disconnection LSM presents the percentage of results map in atlas regions (only regions containing >10% of the result map are shown). The tract-wise LSM presents the partial correlation coefficients. All the regions are in the left hemisphere. **p* < 0.05; ***p* < 0.01; ****p* < 0.001. LSM: lesion symptom mapping; CIU: correct information unit; AF: arcuate fasciculus; FAT: frontal aslant tract; CC: corpus callosum; SLF: superior longitudinal fasciculus; TR_S: superior thalamic radiation; SMG: supramarginal gyrus; PreCG: precentral gyrus.

**Supplementary Table 7. Summary of LSM results of the Western aphasia battery fluency**

|  | White matter | Grey matter |
| --- | --- | --- |
| Lesion LSM | AF 14% | SMG 11%, STG 10%, MTG 12% |
| Disconnection LSM | AF 15%, CC 18%, FAT 12% |  |
| Tract LSM | AF -0.34**, FAT -0.22*, SLF2 -0.29**, SLF3 -0.26* |  |

Lesion/Disconnection LSM presents the percentage of results map in atlas regions (only regions containing >10% of the result map are shown). The tract-wise LSM presents the partial correlation coefficients. All the regions are in the left hemisphere. **p* < 0.05; ***p* < 0.01; ****p* < 0.001. LSM: lesion symptom mapping; AF: arcuate fasciculus; FAT: frontal aslant tract; CC: corpus callosum; SLF: superior longitudinal fasciculus; SMG: supramarginal gyrus; STG: superior temporal gyrus; MTG: middle temporal gyrus.
